# Fms-like tyrosine kinase 3-internal tandem duplications epigenetically activates checkpoint kinase 1 in acute myeloid leukemia cells

**DOI:** 10.1038/s41598-021-92566-5

**Published:** 2021-06-24

**Authors:** Yudong Zhang, Lingli Yuan

**Affiliations:** 1 Department of Critical Care Medicine, The Second Xiangya Hospital, Central South University, 139 Renmin Road, Changsha, 410011 Hunan China; 2 Department of Hematology, The Second Xiangya Hospital, Central South University, 139 Renmin Road, Changsha, 410011 Hunan China

**Keywords:** Cancer, Cell biology, Molecular biology, Oncology

## Abstract

It is not clear how Fms-like tyrosine kinase 3-internal tandem duplications (FLT3-ITD) regulates checkpoint kinase 1 (CHK1) in acute myeloid leukemia (AML). In this study, we investigated the regulatory effect of FLT3-ITD on CHK1. Our results showed that CHK1 was highly expressed in FLT3-ITD positive AML. The overall survival rate and disease-free survival rate of AML patients with high CHK1 level were lower than those of patients with low CHK1 level. Mechanistically, FLT3-ITD recruited p300 to the CHK1 promoter and subsequently acetylated H3K27, thereby enhancing the transcription of CHK1. Interfering with the expression of CHK1 significantly inhibited the cell proliferation and induced cell apoptosis in FLT3-ITD positive MV4-11 cells. In addition, CHK1 knockdown promoted the sensitivity of MV4-11 cells to the epigenetic inhibitors JQ1 and C646. This study discovers a new therapeutic target for FLT3-ITD + AML and provided evidence for the combination of epigenetic inhibitors for AML treatment.

## Introduction

Fms-like tyrosine kinase 3-internal tandem duplications (FLT3-ITD) mutation is the most frequent mutation type (25–30%) in acute myeloid leukemia (AML)^1^. Stone et al. showed that FLT3 inhibitors combined with other drugs effectively improved the prognosis of FLT3 mutant AML patients^2^. FLT3-ITD mutation is one of the independent poor prognostic factors of AML patients and is characterized with easy recurrence and short survival time, and poor efficacy for traditional chemotherapy^3^. Therefore, in-depth study of the pathogenesis of FLT3-ITD^+^ AML will help to provide effective targets for the treatment.

In AML cells, the FLT3-ITD fusion protein with tyrosine kinase activity encoded by the mutation of the FLT3-ITD gene activates the downstream STAT5-PIM, PI3K-AKT and other cell signaling channels, resulting in accelerated proliferation of leukemia cells, thereby inducing DNA replication stress^1^. The mechanism of how FLT3-ITD^+^ AML responds to DNA replication pressure has not been elucidated.

Checkpoint kinase 1 (CHK1) phosphate cascade is currently recognized as the main regulator of the cell cycle when cells respond to exogenous DNA damage. However, few studies have been conducted on how CHK1 regulates endogenous DNA damage (such as DNA replication pressure induced by oncogenes)^4^. Recently, studies have shown that high expression of Chk1 in transgenic mouse models can reduce the pressure of oncogene-induced toxic DNA replication and contribute to tumorigenesis. T-cell acute lymphoblastic leukemia (T-ALL) has a relatively high DNA replication pressure. CHK1 expression and activity in T-ALL cells are higher than in normal cells, and the DNA replication pressure caused by oncogenes phosphorylates the Chk1 cascade pathway, and the activated Chk1 suppresses the DNA replication pressure, thereby avoiding the DNA replication pressure to activate the ATM/caspase-3 apoptosis pathway, which promotes the survival of T-ALL cells^5^. However, the mechanism underlying activated CHK1 in AML remains unclear.

FLT3-ITD has a transcriptional regulation function, but it is not clear whether it has direct regulatory effect on CHK1. In this study, our results elucidate the epigenetic regulatory mechanism of FLT3-ITD on CHK1. CHK1 may be a promising clinical biomarker and therapeutic target for FLT3-ITD-positive AML patients.

## Materials and methods

### Human sample collection

Bone marrow specimens of patients with primary acute myeloid leukemia diagnosed in our hospital (N = 120) were collected from March 2009 to March 2019. The diagnostics of all of them meet the diagnostic criteria of FAB classification. Among them, there were 62 males and 58 females (18–72 years, with an average of 55.4 ± 13.6 years). At the same time, 56 patients with non-hematological tumor diseases who underwent bone marrow aspiration were included as a control group, including 30 males and 26 females (22–70 years, with an average of 52.6 ± 14.2 years). There was no significant difference in age and gender between the two groups (P > 0.05). This study was approved by the Ethic Committee of Second Xiangya Hospital, Central South University. We confirmed that all experiments were performed in accordance with relevant guidelines and regulations. The written informed consent was obtained from each patient.

### Cell culture

Jurkat, MV4-11, THP1, Kasumi-1 cells were purchased from Cell Bank of Chinese Academy of Sciences (Shanghai, China). These cells were cultured in RPMI 1640 medium supplemented with 50 μg/ml streptomycin, 50 IU penicillin and 10% fetal bovine serum.

### Cell transfection

FLT3-ITD was knocked down as the published literature using siRNA sequences directed to the FLT3-ITD mRNA fusion site^6^. For shRNA-mediated knockdown of the CHK1 gene, the shRNA sequence used in this study was synthesized by GenePharma (Shanghai, China) and packaged into the hU6-MCS-PGK-EGFP lentiviral vector (GenePharma, Shanghai, China). Briefly, 1 × 10^6^ MV4-11 cells were seeded in 6-well plates to 80% confluence, and then transfected with shRNA lentivirus with 100 multiplicity of infection. The transfection efficiency was determined by FACScalibur flow cytometry (Becton Dickinson, Franklin Lakes, NJ, USA), and the transfection efficiency exceeded 90%.

### Quantitative polymerase chain reaction (qPCR)

Total RNA was extracted using Trizol reagent. After DNAse I (Thermo Scientific) treatment, RNA was reverse transcribed with reverse transcriptase (Thermo Scientific) to cDNA. QPCR was measured by All-in-One qRT-PCR (GeneCopoeia, Shanghai, China) on the CFX96 real-time PCR detection system (Bio-Rad, Hercules, California, USA) according to the protocol recommended by the manufacturer. Sequence of CHK1 primer (5′– > 3′): Forward primer: CACTCTGCTTCACCGACTGT, Reverse primer: CACCCCTGCCATGAGTTGAT.

### Dual luciferase reporter gene assay

RT-PCR was performed to generate the DNA fragments that was inserted into the pGL3-LUC report vector (Promega). To determine the luciferase activity, 100 ng of blank pcDNA3 vector or pcDNA3 vector containing FLT3-ITD cDNA and 400 ng LUC reporter vector were co-transfected into HEK293T cells for 48 h in a 24-well plate. The cells transfected with pRL-TK Renilla luciferase reporter gene vector (Promega) were used as a control. The luciferase activity was analyzed using the dual luciferase assay (Promega) according to the manufacturer's instructions. The primer sequences were shown in Supplementary Table 1.

### Chromatin immunoprecipitation (ChIP)

A ChIP-seq experiment was performed to determine the protein binding on CHK1 promoter. After knocking down FLT3-ITD in MV4-11 cells, the cells were collected and prepared for cross-linked chromatin. A Bioruptor UCD-200 ultrasound system (Diagenode) was used to break the chromatin into fragments of about 200 bp in size. The fragments were incubated with specific antibodies (anti-CBP (ab 119,488, Abcam), anti-p300 (ab14984, Abcam), anti-H3K9ac (ab272150, Abcam), anti-H3K27ac (ab203953, Abcam) at 4 degrees overnight. Rabbit serum was used as a control. High-Sensitivity ChIP Kit (ab185913, Abcam) was used for RNA elution and purification according to the manufacturer's instructions. QPCR was used to detect the degree of enrichment of the CHK1 promoter sequence.

### Western blot analysis

After treatment, cells were lysed in RIPA buffer containing 1 mM DTT, 11 μg/ml DNase I and protease inhibitor cocktail (Roche), and incubated on ice for 30 min. Sixty micrograms of protein samples were separated by electrophoresis on a denatured 10% SDS-PAGE gel and blotted onto PVDF membranes (Millipore). Immunoblotting was performed using primary antibody (anti-CHK1 (ab40866, Abcam)); rabbit monoclonal antibody to GAPDH (EPR16891, Abcam) was used as a loading control. The protein was visualized by ECL method (Beyotime Biotechnology, Shanghai, China). The original blots were presented in Supplementary Figs. 1 and 2.

### CCK-8 analysis of cell proliferation

For cell proliferation assays, cell proliferation was evaluated using the CCK-8 kit (Beyotime Biotechnology, Shanghai, China) according to the manufacturer's instructions. Briefly, the transfected cells were seeded into 96-well plates (3000 cells/well) and culture was continued for 24 h, and then 10 μl of CCK-8 solution was added to the cell culture. After an additional 4 h of incubation, absorbance was measured at a wavelength of 450 nm.

### Cell apoptosis analysis

The cells were digested with 0.25% trypsin for 3 min. The cells were wash with 1 × phosphate buffer solution (PBS) to eliminate EDTA, so as to avoid chelation of residual EDTA and Ca^2+^, affecting the binding of Annexi V. The cells were collected by centrifuging at 300 × g for 5 min at room temperature. The cells were resuspended with 50 μl of pre-chilled 1 × PBS (4 °C). The cells were mixed gently with labeling solution (10 μl 10 × Binding Buffer, 5 μl annexin V-FITC, 85 μl distilled water), and incubated for 15 min at room temperature in the dark. Each specimen was then added with 10 μl PI for 15 min. Flow cytometry was performed within 30 min.

### Statistical analyses

Graphpad Prism (version 7.04) was used for statistical analysis. The data is expressed as the mean ± standard deviation. Student *t* test was used for two groups comparison. One-way analysis of variance with post-hoc Tukey's test was used for three or more comparisons. Kaplan–Meier method with log-rank test was used for overall survival and disease-free survival analysis. P < 0.05 was statistically significant.

## Result

### Association between CHK1 expression and survival of AML patients

**Figure 1:**
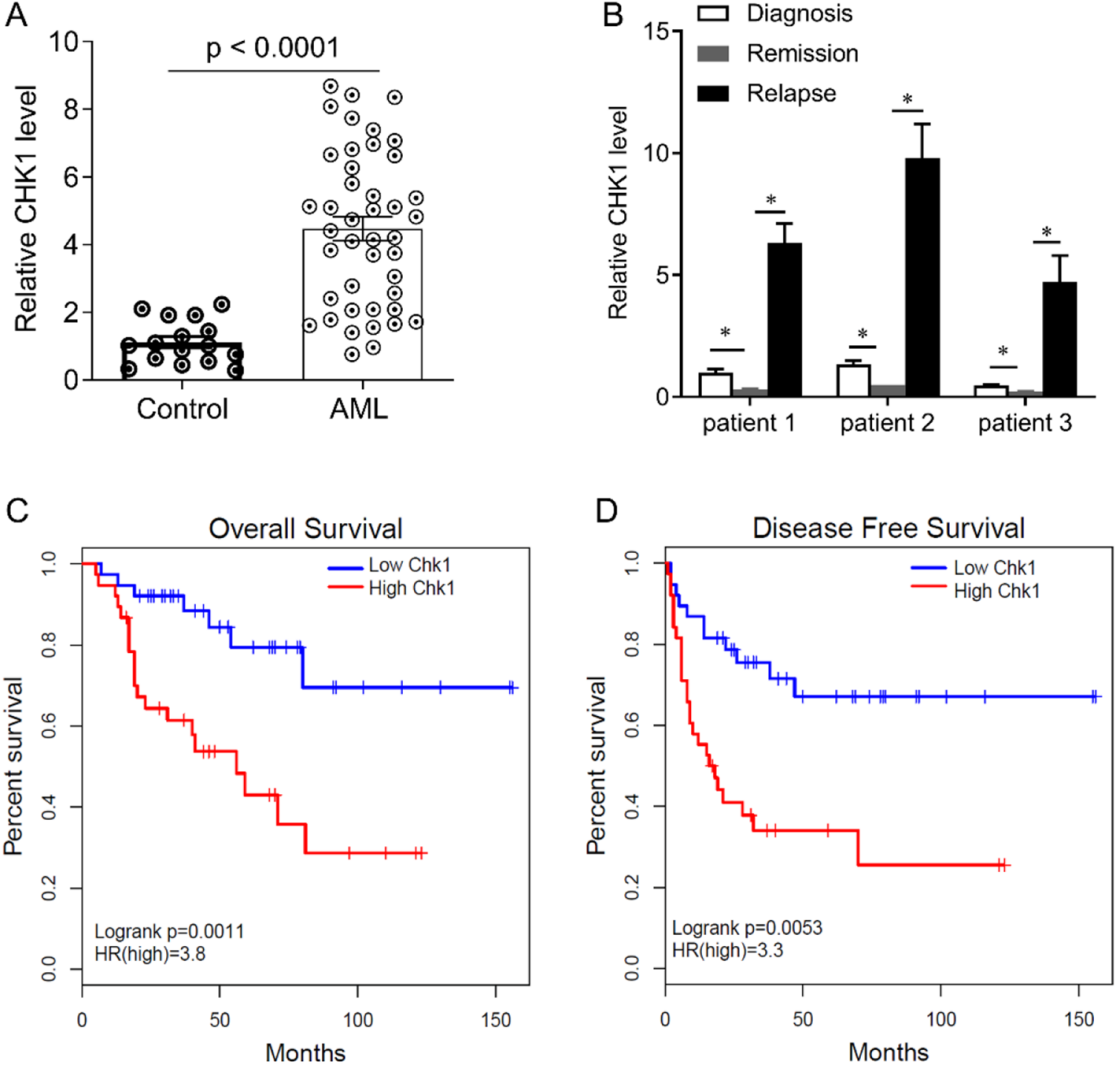
The relationship between the expression of CHK1 and the clinical outcome of AML patients. (**A**) The expression of CHK1 in AML and the control group. (**B**) CD34^+^CD38^−^ cells were isolated from bone marrow samples of AML patients with three different disease stages (including new diagnosis, remission, and relapse), and CHK1 mRNA levels were examined. (**C**) Correlation between the expression of CHK1 and overall survival rate (P = 0.0011, Hazard rate (HR) = 3.8). (**D**) Correlation between CHK1 expression and disease-free survival rate ((P = 0.0053, Hazard rate (HR) = 3.3). The expression value is shown as mean ± SEM. *P < 0.05.

We detected the expression of CHK1 in CD34^+^CD38^−^ cells from bone marrow samples by qPCR. The results showed that the expression of CHK1 in patients with AML was significantly higher than that in the control group (Fig. 1A). In addition, we analyzed the mRNA levels of CHK1 in bone marrow samples of FLT3-ITD^+^ AML patients before and after chemotherapy. The mRNA level of CHK1 was significantly reduced in the remission period compared with in the time of diagnosis, while was significantly increased in the relapse period after chemotherapy (Fig. 1B). We analyzed the relationship between the expression of CHK1 and the prognosis of AML patients. The expression of CHK1 greater than or equal to the average value was defined as high expression, otherwise it was defined as low expression. The AML patients were divided into high expression of CHK1 (n = 71) and low expression of CHK1 (n = 49). The overall survival rate of patients with high CHK1 was lower than that of patients with low CHK1 (Fig. 1C). The disease-free survival rate of patients with high was shorter than that of patients with low CHK1 (Fig. 1D). In summary, these results indicate that the high expression of CHK1 is related to the poor prognosis of AML patients.

### FLT3-ITD epigenetically enhances CHK1 transcription

**Figure 2:**
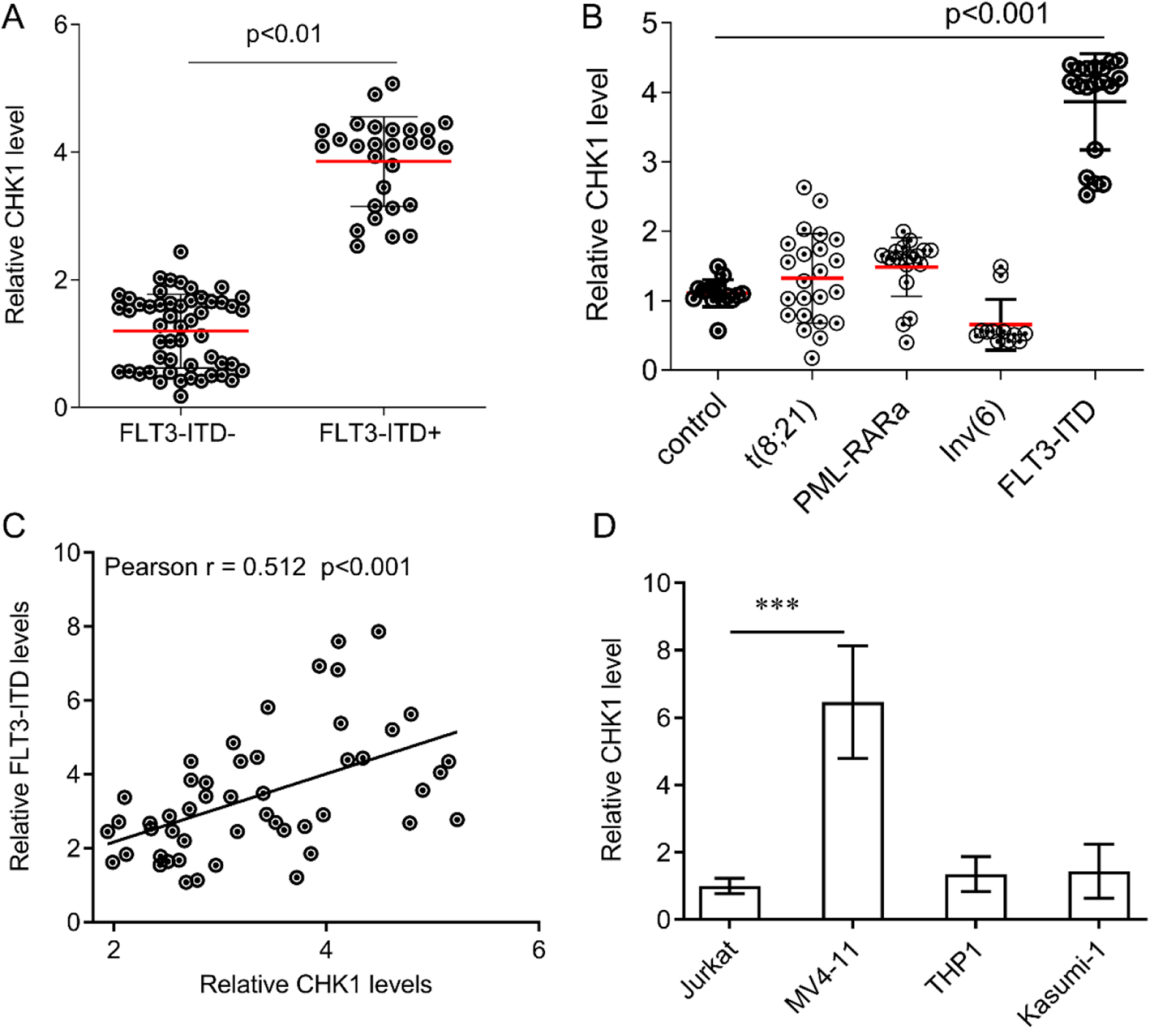
The expression of CHK1 is positively correlated with the expression of FLT3-ITD. (**A**) qRT-PCR was used to detect the expression of CHK1 in CD34^+^CD38^−^ cells in bone marrow samples of FLT3-ITD positive and negative AML patients. (**B**) qRT-PCR was used to detect the expression of CHK1 in CD34^+^CD38^−^ cells from bone marrow samples of t (8; 21), PML-RARa, Inv (16) and FLT3-ITD positive AML patients. (**C**) Correlation of between CHK1 and FLT3-ITD (Pearson test, R = 0.512, p < 0.001). (**D**) Detection of CHK1 expression in AML cell line by qRT-PCR. Data are expressed as mean ± SEM. *p < 0.05.

To investigate the reasons for the upregulation of CHK1 in AML, we analyzed the expression of CHK1 in FLT3-ITD positive and negative AML. We found that the expression of CHK1 in FLT3-ITD positive AML cases was significantly higher than that of FLT3-ITD negative AML cases (Fig. 2A), while there was no significant difference in the expression of other genotypes, including t(8;21), PML-RARa, and Inv(6) (Fig. 2B). By Pearson correlation analysis, we found that the expression of CHK1 in patients with AML was positively correlated with the level of FLT3-ITD (r = 0.512, p < 0.001, Fig. 2C). In the AML cell line, we also found that the expression of CHK1 in FLT3-ITD^+^ AML cells MV4-11 was significantly higher than other FLT3-ITD negative AML cells (Fig. 2D). These results suggest that the upregulation of CHK1 is closely related to the FLT3-ITD mutation.

**Figure 3:**
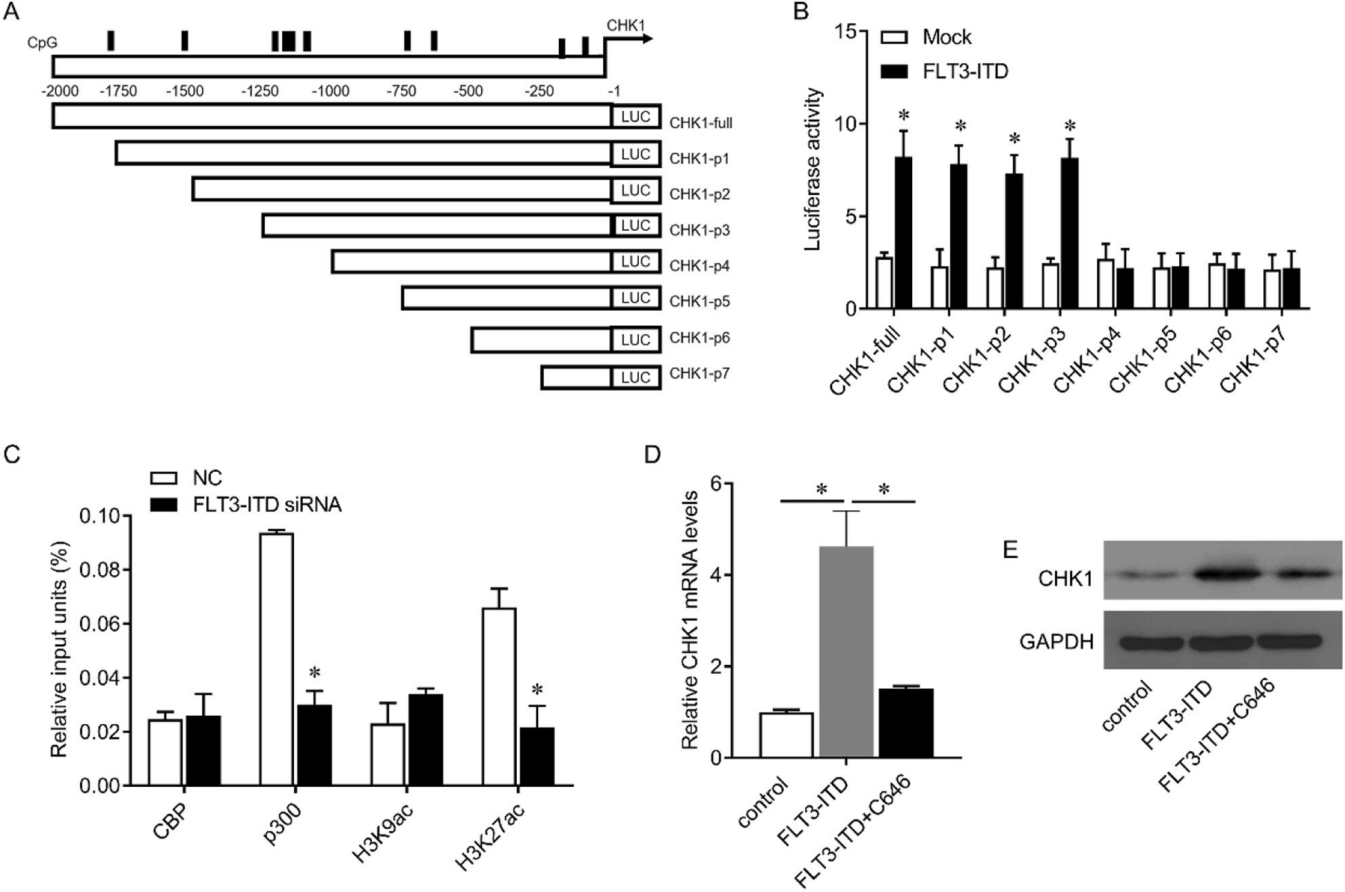
FLT3-ITD epigenetically activates CHK1. (**A**) Schematic representation of the CpG island of the CHK1 promoter. Vertical lines indicate CpG islands (upper panel). Schematic diagram of the construction of the luciferase reporter vector for the full-length fragment of the CHK promoter and its truncated fragment (every 250 bases). The numbers indicate the nucleotide position relative to CHK1 (+ 1 nt) (low panel). (**B**) 293 T cells were co-transfected with CHK1 promoter luciferase reporter vector and FLT3-ITD expression vector for 48 h, the luciferase reporter activity was detected. (**C**) After transfection of FLT3-ITD siRNA in MV4-11 cells, ChIP was performed using the designated antibody or IgG, and qRT-PCR was performed to detect the enrichment of protein on the CHK1 promoter. (**D**, **E**) Kasumi-1 cells were transfected with FLT3-ITD expression vector and treated with C646 (30 nM) for 24 h. qRT-PCR (**D**) and Western blot (**E**) were used to detect the expression of CHK1 mRNA and protein. The original blots were presented in Supplementary Fig. 1. The expression value is shown as the mean ± SEM. *p < 0.05.

To examine how FLT3-ITD regulates CHK1, the methylation sites of the promoter region of CHK1 and possible FLT3-ITD binding sites were analyzed by bioinformatics (Fig. 3A). We constructed a luciferase reporter gene, which contains the full-length type of CHK1 promoter region and a serial of truncated fragments (CHK1-full, CHK1-P1-P7). Each reporter gene and FLT3-ITD or empty vector were co-transfected into 293 T cells to detect luciferase activity. The results showed that FLT3-ITD overexpression significantly enhanced CHK1-full, CHK1-P1-P3 luciferase activity, but did not increase CHK1-P4-P7 luciferase activity (Fig. 3B), indicating that the binding region of FLT3-ITD on CHK1 promoter was at 1000–1250 nt upstream the transcription start site and may be involved in FLK3-ITD-induced CHK1 expression.

Through ChIP experiment, we further analyzed whether FLT3-ITD can recruit epigenetic regulatory molecules to the promoter region of CHK1. The results showed that interfering with FLT3-ITD significantly reduced the enrichment of p300 in the CHK1 promoter region, but had no significant effect on CBP, while significantly reducing the acetylation level of H3K27, indicating that FLT3-ITD can recruit p300 to the promoter of CHK1 and acetylates H3K27, thereby activating CHK1 transcription (Fig. 3C). To further demonstrate this effect, we expressed exogenous FLT3-ITD in Kasumi-1 cells that were negative for FLT3-ITD, and treated Kasumi-1 cells with p300 small molecule inhibitor C646. Exogenous FLT3-ITD significantly increased the expression of CHK1 mRNA and protein, and this increase can be significantly reduced by C646 treatment (Fig. 3D, E). Therefore, these results indicate that FLT3-ITD recruits p300 to the CHK1 promoter and activates CHK1 transcription.

### The effect of CHK1 on FLT3-ITD positive AML cells

**Figure 4:**
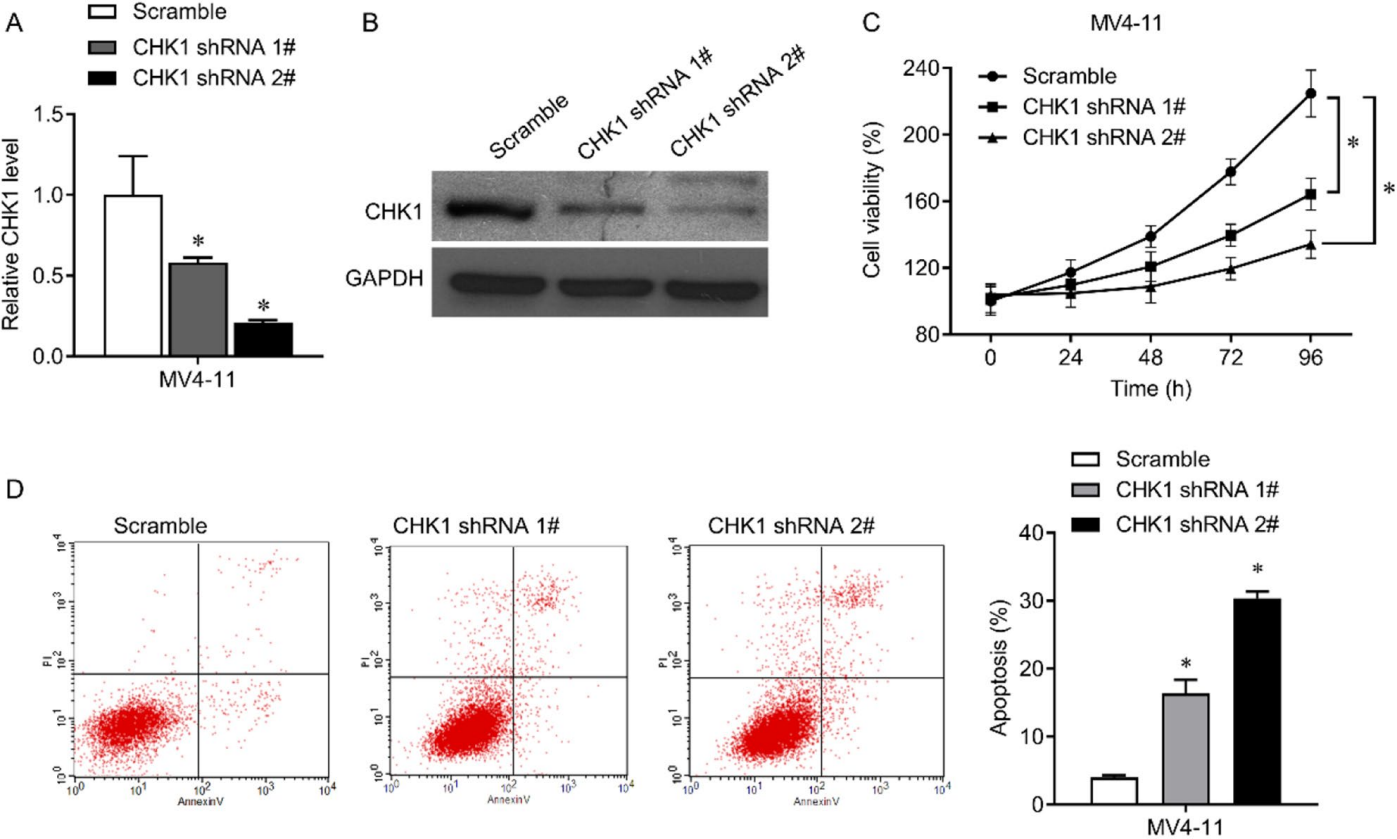
The effect of shRNA-mediated CHK1 knockdown on FLT3-ITD-positive AML cells. (**A**,**B**) 48 h after infection of MV4-11 cells with shRNA lentivirus, qRT-PCR (**A**) and Western blot (**B**) were used to detect the expression of CHK1. The original blots were presented in Supplementary Fig. 2. (**C**) CCK8 assay measured the cell viability of MV4-11 cells after treatment. (**D**) The effect of CHK1 knockdown on cell apoptosis in MV4-11 cells. The expressed values are shown as mean ± SEM. *p < 0.05.

We further analyzed the function of CHK1 in AML. We designed two shRNA sequences to reduce the expression of CHK1. Transfecting shRNAs in MV4-11 cells can significantly reduce CHK1 mRNA expression and protein levels, while shRNA 2 # was more efficient (Fig. 4A,B). Compared with the negative control, the interference with CHK1 significantly reduced the cell activity of MV4-11 cells (Fig. 4C) and promoted the apoptosis of MV4-11 cells (Fig. 4D). Therefore, downregulation of CHK1 can inhibit the proliferation of FLT3-ITD positive AML cells.

### Interfering with CHK1 enhances the sensitivity of FLT3-ITD-positive AML cells to epigenetic inhibitors

**Figure 5:**
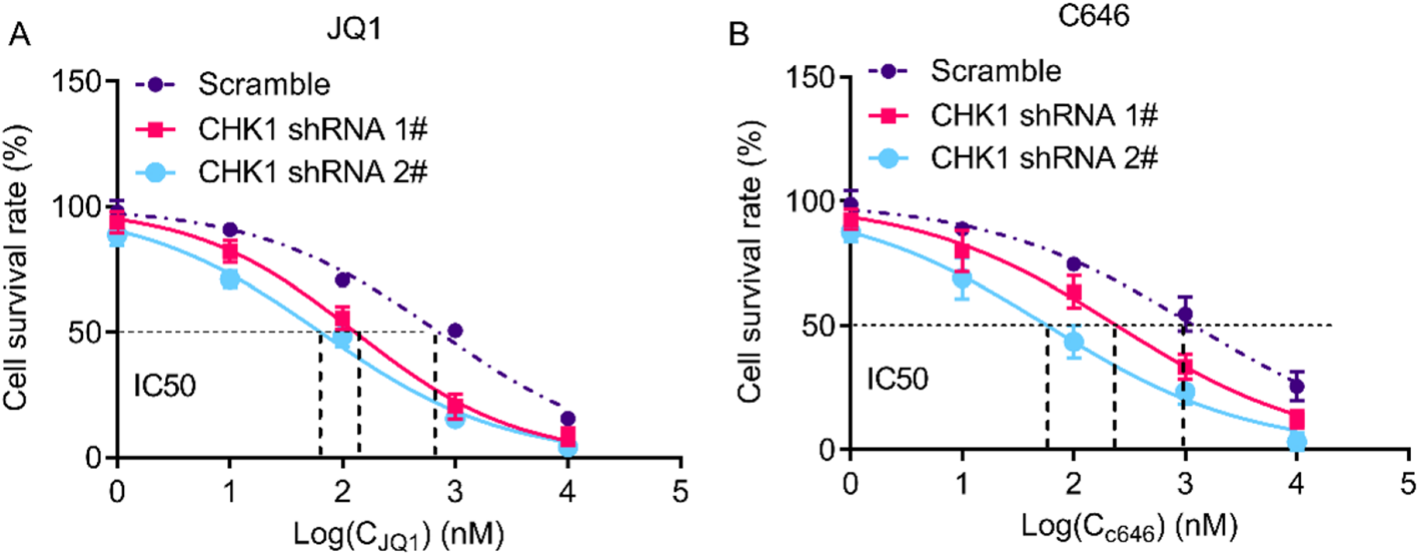
CHK1 knockdown increases the sensitivity of MV4-11 cells to epigenetic inhibitors. (**A**) MV4-11 cells transfected with CHK1 shRNA were treated with increasing concentrations of JQ1 for 24 h. CCK8 assay measured the cell viability. (**B**) MV4-11 cells transfected with CHK1 shRNA were treated with increasing concentrations of C646 for 24 h. CCK8 assay measured the cell viability.

In order to further evaluate the role of CHK1 in AML chemotherapy, we simultaneously knocked out CHK1 and treated the MV4-11 cells with epigenetic inhibitors JQ1 and C646. The results showed that CHK1 knockdown significantly increased the sensitivity of MV4-11 cells to JQ1 and C646, and significantly reduced the half inhibitory concentration (IC50) of JQ1 (Control: 715.6 nM, shRNA1 #: 129.7 nM, shRNA2 #: 63.8 nM ) and the IC50 of C646 (Control: 1202 nM, shRNA1 #: 244.7 nM, shRNA2 #: 58.1 nM) (Fig. 5). These results indicate that targeting CHK1 in combination with epigenetic inhibitors can improve the therapeutic effect of FLT3-ITD-positive AML.

## Discussion

In this study, we clarified that FLT3-ITD positively regulated CHK1 through epigenetic mechanisms, thereby promoting AML cell proliferation. Interfering with the expression of CHK1 significantly inhibited proliferation and promoted apoptosis in FLT3-ITD positive AML cells, and increased the sensitivity of AML cells to epigenetic inhibitors. This study provided a new therapeutic target for FLT3-ITD positive AML treatment and provided a basis for the combination of epigenetic inhibitors to treat AML.

Tyrosine kinase helps promote cell proliferation and differentiation. The mutation of FMS-like tyrosine kinase-3 gene (FLT3) is closely related to the occurrence and development of AML disease^7^. FLT3 is regarded as an oncogene that causes potential leukemia^8^. It locates in the cell membrane and is a stimulating growth factor receptor^9^. Mutation of FLT3 gene causes abnormal differentiation of normal bone marrow CD34 + hematopoietic stem/progenitor cells^10^. FLT3-ITD is the main type of gene mutation in internal tandem replication (ITD) located near the membrane region, accounting for 20% to 35%. Patients with FLT3-ITD mutation-positive AML tend to have more severe disease, lower remission rates, and poor survival rates^11^. In this study, we found that CHK1 directly interacted with FLT3-ITD. In addition, the expression of CHK1 was positively correlated with FLT3-ITD expression, whereas was negatively correlated with overall survival and disease-free survival time. We need a larger sample to confirm this conclusion.

FLT3 mutation regulates the differentiation and proliferation of cells in the bone marrow by binding multiple proteins^12,13^. FLT3-ITD recruits methyltransferase on the promoter to silence the target gene, but recruits acetylation coactivator to activate the transcription of the target gene^14^. Exogenous expression of FLT3-ITD induces Rac1 activity, phosphorylation of STAT5, increased DNA damage response factor and cytarabine resistance^15^. In the mouse model of FLT3-ITD AML, animals treated with CHK1 inhibitor MK8776 plus cytarabine survived longer than animals treated with cytarabine alone^16^. Inhibition of FLT3-ITD by sorafenib downregulates DNA damage-induced Chk1 prominently enhances induction of apoptosis^17^. A high abundance of CHK1 in AML patient cells correlated with higher clonogenic ability and more efficient DNA replication fork progression upon cytarabine treatment^18^. Our previous study reported that pharmacological inhibition of CHK1 and shRNA-mediated down-regulation reduced the proliferation rate of FLT3-ITD expressing leukemic cells in a cytostatic manner. CHK1 overexpression increased the proliferation rate of FLT3-ITD-expressing cells^19^. In this study, luciferase reporter gene assay showed that FLT3-ITD bound to the −1250–1000 region in the CHK1 promoter. In addition, we found that FLT3-ITD enhanced transcription by recruiting p300 to the promoter of CHK1 and acetylating H3K27. The suppression of CHK1 expression by treatment with histone acetylation inhibitor C646 also confirmed this hypothesis. These findings suggest that the addition of DNA damage response inhibitors to conventional chemotherapy may be useful in the treatment of FLT3-ITD AML. However, in other subtypes of AML, it is unclear whether other fusion genes directly regulate the expression of CHK1, and further research is needed.

This study clarified the function of CHK1 in FLT3-ITD-positive AML. Interfering with CHK1 significantly inhibited the proliferation of AML cells. CHKl protein kinase is highly expressed in many tumors such as breast cancer, colon cancer, liver cancer, gastric cancer, and nasopharyngeal cancer, but because of its key role in cell cycle checkpoints, it is initially considered as a tumor suppressor ^20^. However, with the deepening of the research, the researchers found that the ATR-CHKl axis and CHKl protein kinase promote the growth of tumor cells. The tumor cells with high expression of CHK1 have more survival advantages than the tumor cells with low expression^21^. The cells with higher CHKl protein kinase can better adapt to the severe tumor microenvironment or the DNA damage response caused by radiotherapy and chemotherapy, so eventually lead to the generation of drug-resistant clones and tumor recurrence^22^. CHKl protein kinase inhibitors are currently used as a sensitizer in clinical research. It can be used in combination with chemotherapy drugs to increase the sensitivity of tumor cells to chemotherapy, thereby reducing their adverse reactions and increasing efficacy^23^. Studies have shown that the CHK1 inhibitor MK-8776 can significantly increase the sensitivity of leukemia cells to histone deacetylase inhibitors by disrupting the mechanisms of DNA replication and DNA repair, including FLT3-ITD + leukemia cells^16^. Previous study showed that ectopic expression of CHK1 improved the resistance of FLT3-ITD cells and maintained histone H3 phosphorylation in response to DNA damage^24^. It can be used in combination with other molecular targeted drugs to produce a "synthetic lethal" effect. For example, Wang et al. found that All-trans retinoic acid (ATRA) down-regulated Chk1 in FLT3-ITD AML cells, and the combination of ATRA and DNA damage drug SN38 significantly improved the anti-tumor effect of either ATRA or SN38 when used alone^25^.

In addition, the combination of CHK1 silencing and epigenetic inhibitors JQ1 and C646 significantly enhanced the inhibition of AML cell proliferation. JQ1 is an inhibitor of the BET family and can compete with acetylated histones for binding to the BET-derived bromodomain (BD), thereby inhibiting BRD4 from recruiting P-TEFb to the promoter region of the gene, thereby inhibiting transcription^26^. Due to the efficiency in treatment of leukemia, colon cancer and prostate cancer, JQ1 is considered as a potential effective drug for clinical treatment of cancer^27^. JQ1 treatment resulted in a rapid, time-dependent reduction in CHK1 phosphorylation and aberrant DNA replication re-initiation^28^, and enhanced the cytotoxicity of CHK1 inhibition in ovarian cancer^29^. This study found that FLT3-ITD increased the expression of CHK1 by enhancing the acetylation of the CHK1 promoter. The synergistic relationship between CHK1 interference and epigenetic inhibitors in FLT3-ITD-positive AML has opened a new way for the development of AML drugs.

In conclusion, this study shows that FLT3-ITD activates CHK1 through an epigenetic mechanism, thereby promoting AML progression. These findings also indicate that CHK1 is a promising biomarker and therapeutic target for FLT3-ITD + AML.

## Supplementary Information

Supplementary Information.

## Data Availability

All data generated or analysed during this study are included in this published article.
