# Supplementary material for: Fms-like tyrosine kinase 3-internal tandem duplications epigenetically activates checkpoint kinase 1 in acute myeloid leukemia cells
Source: Sci Rep. 2021 Jun 24;11:13236. doi: 10.1038/s41598-021-92566-5 (PMC8225911; doi:10.1038/s41598-021-92566-5)
Supplement: Supplementary file 1 — Supplementary Information. [file 41598_2021_92566_MOESM1_ESM.pdf]

Fms-like tyrosine kinase 3-internal tandem duplications epigenetically activates checkpoint kinase 1 in acute myeloid leukemia cells

Running title: FLT3-ITD epigenetically activates CHK1

Yudong Zhang[1], Lingli Yuan[2*]

1, Department of Critical Care Medicine, The Second Xiangya Hospital, Central South University,

2, Department of Hematology, The Second Xiangya Hospital, Central South University, Changsha, Hunan 410011, China

*Corresponding author: Yuan Lingli, Department of Hematology, The Second Xiangya Hospital, Central South University

Address: 139 Renmin Road, Changsha, Hunan, 410011, China

Email: lingliestelle@csu.edu.cn

supplementary Table 1 Primer sequences used for dual luciferase reporter gene assay

CHK1 promoter fragments	forward (5'->3')	reverse (5'->3')
CHK1-full	TATTTTGGGAGGTTGAAGTATGC	AAATTACAAACATAATCCACTACGC
CHK1-P1	TTTTAAGAGTGGGATTTTAGGTAGG	ATTACAAAATATATACCCCACACCC
CHK1-P2	AAATTTTTGTTAGTTTGTTTTTTTT	AAAATATATACCCCACACCCAACTA
CHK1-P3	AAATTACACGATCTATACCCCACATT	AATATATACCCCACACCCAACTAATT
CHK1-P4	GAGCTCCCAGGAACCAATGT	ACTTGCGGAAAGGATGGGTT
CHK1-P5	TTACAAAATATATACCCCACACCC	AAATTACAAACATAATCCACTACACC
CHK1-P6	TATTTTGGGAGGTTGAAGTATGTG	AATTACAAACATAATCCACTACACC
CHK1-P7	TAAAATTACAAACATTCCACTACCG	AAAATTACAAACATAATCCACTACGC

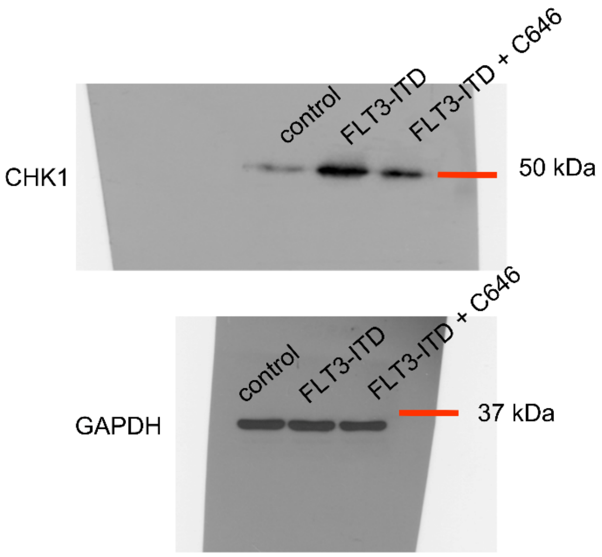

Supplementary Figure 1 The original blots of CHK1 and GAPDH in Figure 3E. Western blot was performed to test the expression of CHK1 after indicated treatment.

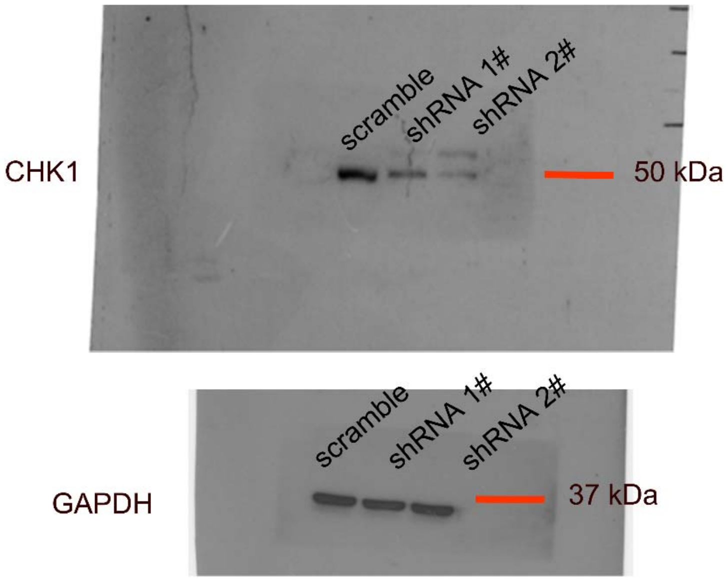

Supplementary Figure 2 The original blots of CHK1 and GAPDH in Figure 4B. Western blot was performed to test the expression of CHK1 after shRNA transfection.
